# Unilateral nasal obstruction: a rare presentation of acute myeloid leukaemia

**DOI:** 10.1093/jscr/rjab581

**Published:** 2021-12-28

**Authors:** Mairead Kelly, Rajeev Advani, James Schuster-Bruce, Eleanor Crossley, Raj Lakhani

**Affiliations:** Department of Ear, Nose and Throat Surgery, St George’s University Hospitals NHS Foundation Trust, London, UK; Department of Ear, Nose and Throat Surgery, St George’s University Hospitals NHS Foundation Trust, London, UK; Department of Ear, Nose and Throat Surgery, St George’s University Hospitals NHS Foundation Trust, London, UK; Department of Ear, Nose and Throat Surgery, St George’s University Hospitals NHS Foundation Trust, London, UK; Department of Ear, Nose and Throat Surgery, St George’s University Hospitals NHS Foundation Trust, London, UK; Department of Ear, Nose and Throat Surgery, Epsom and St Helier University Hospitals NHS Trust, Epsom, UK

**Keywords:** acute myeloid leukemia, myeloid sarcoma, nasal obstruction, otolaryngology, diagnostic techniques and procedures

## Abstract

Myeloid sarcoma, and, with it, Acute Myeloid Leukaemia (AML), is a rare but important differential diagnosis in the consideration of unilateral nasal blockage. These lesions are often misdiagnosed as lymphoma or poorly differentiated carcinoma. We report the case of a patient with unilateral nasal blockage who underwent Endoscopic Sinus Surgery and biopsy. Histology revealed myeloid sarcoma and she was diagnosed with AML. Genetic testing could not be fully undertaken as the biopsy samples were preserved in formalin, which can degrade the quality of the DNA required for the more sensitive fms-like tyrosine kinase 3-internal tandem duplication (FLT3 ITD) test. Given that these levels have a significant impact on treatment decisions, a further biopsy, preserved in saline, was required. This case exemplifies the need for Ear, Nose and Throat clinicians to have a high index of suspicion for this lesion, and a working knowledge of the testing requirements for samples taken.

## INTRODUCTION

A diagnosis of acute myeloid leukaemia (AML) usually requires identification of myeloid blasts in peripheral blood or bone marrow. However, isolated, extramedullary AML—called myeloid sarcoma, granulocytic sarcoma or chloroma—also occurs [1, 2]. A myeloid sarcoma, as reported in this case, is essentially a tumour composed of leukaemic cells [3]. Treatment of AML is informed by rapid, targeted genomic analysis as well as the patient’s performance status [1, 4].

A literature review showed only seven previous case reports of nasal obstruction associated with AML [3, 5–11]. We present a case which elucidates the importance of suspecting AML as a cause of unilateral nasal obstruction not only to expedite diagnosis but also to select appropriate investigative methods.

## CASE REPORT

A 65-year-old female presented with a 12-month history of gradual right-sided nasal obstruction and epiphora. She had a painless lump on the right lateral aspect of her nose. She is an ex-smoker, with a two pack-year smoking history, and drinks alcohol rarely. There were no B-type symptoms.

Her past medical history includes hypertension, mild angina and hypercholesterolaemia. Her World Health Organization performance status is 0.

On examination, the right globe was proptosed and laterally displaced, with a firm mass in the medial canthal area. Her right tear lake was raised. Her visual acuity was found to be unaffected and she had full extraocular movements.

Endoscopic examination revealed a mass within the right nasal cavity, the origin of which could not be visualized. The postnasal space could not be accessed from the right side, but when examined from the left side, was found to be clear. There was a palpable right level 1B neck node. Ear, nose and throat (ENT) examination was otherwise unremarkable.

**Figure 1:**
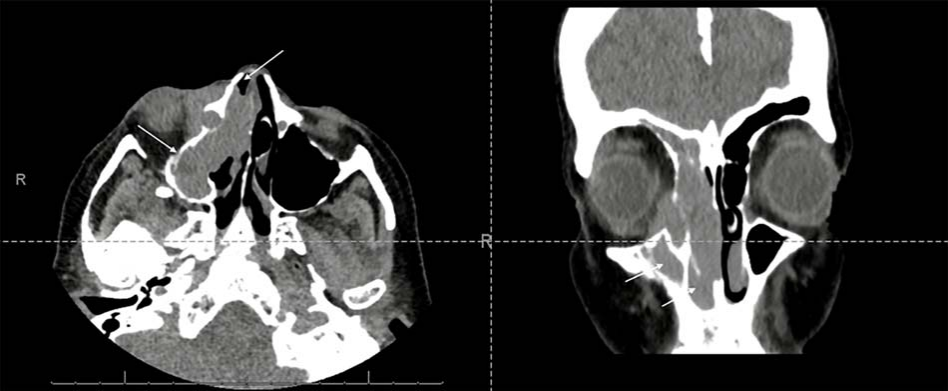
Axial and coronal CT sinus images showing an extensive soft tissue mass within the right maxillary sinus which completely obliterates the sinus cavity and extends into the right nasal cavity (arrows). It also involves the right ethmoid and frontal sinuses. The mass extended through the lamina papyracea into the right orbit, abutting and displacing the globe, and is seen to invade the nasolacrimal system.

Fine-needle aspiration of a right neck lymph node showed reactive hyperplasia, with no evidence of dysplasia or malignancy. Computerized tomography (CT) imaging showed an extensive soft tissue mass within the right maxillary sinus, extending into the right nasal cavity and orbit (Fig. 1).

She underwent endoscopic sinus surgery. Biopsies were taken from the right inferior turbinate, lateral nasal wall, lacrimal fossa and maxillary sinus. As per standard protocols, these were preserved in formalin and submitted for histological analysis. Histology showed myeloid sarcoma.

Positron emission tomography-computed tomography (PET-CT) was performed for staging. This identified the right-sided sino-nasal/orbital mass and small volume right neck nodes. There were no other sites of disease detected. Bone marrow aspirate showed no evidence of marrow involvement. There were no features to suggest antecedent chronic neutrophilic leukaemia or atypical chronic myeloid leukaemia (CML).

Genotyping of the tissue showed mutations in NPM1, as well as CSF3R, IDH1, NF1 and NFE2. It was not possible to test for levels of FLT3 ITD as the original biopsy samples were sent in formalin, which can degrade the quality of the DNA required for this more sensitive test. Given that these levels have a significant impact on treatment decisions, a repeat biopsy was undertaken, 8 weeks after the first. This showed no evidence of a FLT3 ITD. The need for a repeat biopsy delayed decisions about final treatment by ~2 weeks. The patient was counselled that she had a highly malignant condition and the decision was made to treat her with intensive chemotherapy.

The patient has now completed two cycles of chemotherapy. After 3 days, the mass on the side of her nose, as well that in the medial canthal area, had clinically disappeared. Several weeks later, her nasal blockage is completely resolved. A PET-CT following the first cycle of chemotherapy showed a partial response with a reduced metabolic activity and reduction in size of the right sino-nasal mass and the right cervical node.

## DISCUSSION

Myeloid sarcoma (and, with it, AML) is a rare but important differential diagnosis in the consideration of unilateral nasal blockage. Prompt diagnosis and genotyping is critical to successful treatment and requires samples preserved in saline as well as formalin, which is not standard practice for biopsies of nasal masses [5, 6].

The challenge for clinicians managing cases of unilateral nasal blockage is that—macroscopically and radiologically—there is a wide differential diagnosis, and therefore, it is unclear when to use saline preservation. For example, in this case, the radiological diagnoses include fungal sinusitis, benign sino-nasal tumours (such as inverted papilloma) and malignant sino-nasal tumours (such as squamous cell carcinoma or adenocarcinoma). In terms of haematolymphoid tumours, the differential diagnoses include non-Hodgkin Lymphoma, diffuse large B-cell lymphoma, extramedullary plasmacytoma, histiocytic sarcoma and Langerhans cell histiocytosis [3]. Indeed, the literature shows that myeloid sarcomas are often misdiagnosed for lymphoma or poorly differentiated carcinoma [11, 12].

Therefore, to improve the time to treatment of myeloid sarcoma, ENT clinicians should preserve biopsy samples in saline, as well as formalin, at the time of the first biopsy, especially in cases that are radiologically or macroscopically suspicious for a haematolymphoid tumour. Saline preservation allows genotyping for the FLT3 gene. Mutations in these genes are incorporated into the consensus risk stratification guidelines for AML and have prognostic importance, as well as significance for treatment selection [4]. In the case of FLT3 mutation, an FLT3 inhibitor (for example, midostaurin) is added to the chemotherapy regime. Further feasibility and cost-effectiveness studies are required before the adoption of widespread saline preservation in these cases, so as to not overwhelm pathology departments in the context of a rare disease. A literature review revealed only seven case reports of chloroma presenting as a nasal cavity mass or obstruction, ranging from 2001 to 2018 and reporting on both children and adults. In only three of these cases was nasal obstruction the initial presenting symptom of AML, as it was in this case; the other cases all described patients who already had a diagnosis of AML.

Further suspicion for myeloid leukaemia is raised based on its risk factors. These include smoking, high body mass index and occupational exposure to benzene and formaldehyde [13]. In ~10% of those diagnosed with AML, there is exposure to previous cytotoxic chemotherapy, or ionizing radiation, usually as treatment for a primary malignancy [14]. In the literature review, there were also cases of the development of myeloid sarcoma in patients with pre-existing CML or AML—so this is also a vital part of the patient history [3, 7].
